# Assessing the Discriminatory Capabilities of iEK Devices under DC and DC-Biased AC Stimulation Potentials

**DOI:** 10.3390/mi14122239

**Published:** 2023-12-14

**Authors:** Nuzhet Nihaar Nasir Ahamed, Carlos A. Mendiola-Escobedo, Victor H. Perez-Gonzalez, Blanca H. Lapizco-Encinas

**Affiliations:** 1 Microscale Bioseparations Laboratory, Biomedical Engineering Department, Rochester Institute of Technology, 160 Lomb Memorial Drive, Rochester, NY 14623, USA; nn5878@rit.edu; 2 School of Engineering and Sciences, Tecnologico de Monterrey, Monterrey 64849, Mexico; carlos_mendiola2000@hotmail.com

**Keywords:** electrokinetics, electroosmosis, electrophoresis, separation, electropherogram

## Abstract

There is a rising need for rapid and reliable analytical methods for separating microorganisms in clinical and biomedical applications. Microscale-insulator-based electrokinetic (iEK) systems have proven to be robust platforms for assessing a wide variety of microorganisms. Traditionally, iEK systems are usually stimulated with direct-current (DC) potentials. This work presents a comparison between using DC potentials and using DC-biased alternating-current (AC) potentials in iEK systems for the separation of microorganisms. The present study, which includes mathematical modeling and experimentation, compares the separation of bacterial and yeast cells in two distinct modes by using DC and DC-biased AC potentials. The quality of both separations, assessed in terms of separation resolution ($R_{s}$), showed a complete separation ($R_{s}$ = 1.51) with the application of a DC-biased low-frequency AC signal but an incomplete separation ($R_{s}$ = 0.55) with the application of an RMS-equivalent DC signal. Good reproducibility between experimental repetitions (<10%) was obtained, and good agreement (~18% deviation) was observed between modeling and experimental retention times. The present study demonstrates the potential of extending the limits of iEK systems by employing DC-biased AC potentials to perform discriminatory separations of microorganisms that are difficult to separate with the application of DC potentials.

## 1. Introduction

There is a growing need for rapid and reliable methods for the analytical separation of microorganisms in clinical analysis, food safety, and environmental monitoring assessments [1]. There is a plethora of well-established traditional separation techniques for the separation of nanosized analytes (e.g., macromolecules), such as capillary electrophoresis (CE) and liquid chromatography (LC), which are customizable to different target analytes [2,3]. However, there is a lack of well-developed and customizable methods for separating micron-sized analytes, such as microorganisms [1]. Microscale electrokinetic (EK) methods offer an attractive option for analyzing and separating microorganisms due to their attractive characteristics, such as low sample requirements, low cost, high resolution, robustness, and ease of optimization [4,5]. Insulator-based EK (iEK) microfluidic devices have proven to be efficient platforms for assessing a wide range of microorganisms, ranging from viruses to mammalian cells [6]. The presence of three-dimensional (3D) insulating posts or structures within a microchannel distorts the electric field distribution in the iEK device, forming zones of higher electric field intensity and giving rise to nonlinear EK phenomena [7]. Therefore, these systems have the added advantage of combining linear and nonlinear EK effects within the same system, which can be strategically manipulated for separating complex mixtures [7].

The assessment and separation of intact microorganisms with CE systems have been investigated by several research groups, including the Armstrong [8,9,10,11,12], Horká [13,14,15,16,17], and Buszewski [18,19,20,21,22] groups. Intact microbes have also been analyzed with microfluidic iEK systems; for example, the Hayes group reported high-resolution separations of closely related microbial strains [23,24,25]. Our group reported the separation of viruses, bacterial cells, and yeast cells [7]. However, all these separations utilized direct-current (DC) voltages. There are only a few studies that report the separation of microorganisms by applying alternating-current (AC) voltages [26,27,28,29], such as studies by the Ros group [26,27,28], the Xuan group [30,31], and our group [29]. The Morgan group [32,33] has also developed iEK systems stimulated with low-frequency AC potentials, but they separated microparticles only, not microorganisms. However, despite the availability of reports illustrating the separation of microorganisms with the two types of stimulation (i.e., DC-only vs. DC-biased AC potentials), a baseline comparison has not been performed, necessitating further investigation.

Until recently, dielectrophoresis (DEP) was considered to be the major, dominant EK phenomenon in iEK systems stimulated with DC or low-frequency (<1 kHz) AC potentials [34]. Although nonlinear electrophoresis (EP_NL_) was first reported in the 1970s by Dukhin [35], the lack of experiments on EP_NL_ hampered its application [36]. Therefore, the majority of iEK studies ignored the effects of EP_NL_, leading to inaccurate interpretations and prompting the use of correction factors in mathematical models to match experimental results [37]. Recent reports have highlighted the significant effects of EP_NL_ on particle electromigration in iEK systems that have been used for differentiating microparticles and cells [38,39,40,41,42]. Our group considered the EP_NL_ effect in recent reports on the separation of microparticles and cells with similar characteristics by applying either DC or low-frequency AC signals [7,43]. However, none of these studies included a comparison between separations carried out with DC potentials and separations carried out with AC potentials.

The present study addresses this knowledge gap by demonstrating the separation of microorganisms by employing two types of signals—a DC signal and a low-frequency DC-biased AC signal, of which the DC signal was designed to be equivalent in magnitude to the root mean square (RMS) of the DC-biased AC voltage. The two separations presented here employed a binary mixture of microorganisms, *Escherichia coli* (*E. coli*) and *Saccharomyces cerevisiae* (*S. cerevisiae*), which are cells of two distinct domains: prokaryotic and eukaryotic, respectively. To the best of our knowledge, this is the first report comparing the performance of DC-stimulated and DC-biased AC-stimulated iEK separation of microorganisms, where the DC potential is equivalent to the RMS value of the DC-biased AC potential. This work included both numerical modeling with COMSOL Multiphysics version 5.6 and experimentation. The quality of these separations was compared by assessing the electropherograms in terms of separation resolution ($R_{s}$). The experimental results indicated that separation resolution values of $R_{s}$ = 0.55 and $R_{s}$= 1.51 were obtained by applying the DC and the DC-biased AC potentials, respectively. These findings illustrate the added advantage of using AC voltages, which enable separations that are not possible using DC voltages. AC potentials have extra characteristics (frequency, amplitude, and magnitude of the DC bias) that can be modified or customized to enable a desired separation process. Good reproducibility between experimental repetitions, ranging from 2 to 8%, was obtained. The deviations between mathematically predicted and experimental retention times ranged from 5.5 to 18.2%, indicating that the model is a valuable tool for guidance in the design of separation processes. Joule heating was not considered in this study based on our previous publication [44], where no significant heating occurred. These results demonstrate the ability of AC-stimulated iEK systems to separate microorganisms by offering extra parameters that can be tuned to achieve separations that are not possible employing a DC voltage.

## 2. Theory

**Figure 1:**
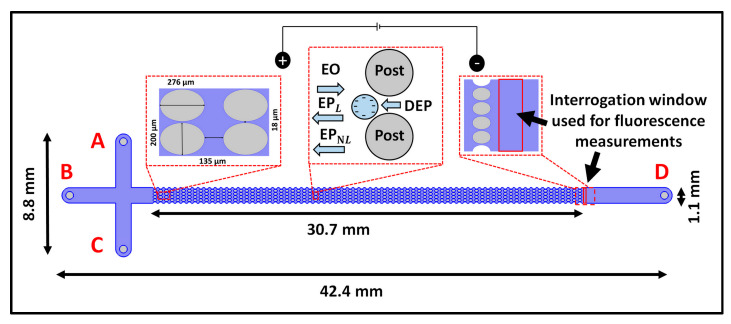
Schematic illustration of the iEK microchannel with four reservoirs labeled A–D, depicting the channel dimensions and the location of the interrogation window used for measuring fluorescence. The first figure inset shows the post dimensions. The second figure inset illustrates the four EK forces (EO, EP_L_, EP_NL_, and DEP) acting on the cells, which possess a negative surface charge and feature a smaller complex permittivity than the suspending solution. The third figure inset indicates the interrogation widow used for fluorescence measurements of the cells eluting from the post array and the walls of the microchannel.

EK phenomena are classified as linear or nonlinear according to their dependence on the electric field. The linear EK phenomena considered here are electroosmosis (EO) and linear electrophoresis (EP_L_), whose velocities, given by $E=E{\hat{a}}_{E}$ (where ${\hat{a}}_{E}$ is a unit vector with the direction of vector E, having a magnitude of E), can be expressed as [43]:

(1) $$v_{EO}=\mu_{EO}E=-\frac{\varepsilon_{m}\zeta_{W}}{\eta }E$$

(2) $$v_{EP,L}=\mu_{EP,L}E=\frac{\varepsilon_{m}\zeta_{P}}{\eta }E\;(\mathrm{weak}\;\mathrm{field}\;\mathrm{regime})$$

where v is the velocity; $\mu_{EO}$ and $\mu_{EP,L}$ are the linear EO and EP mobilities, respectively; $\varepsilon_{m}$ and η denote the permittivity and viscosity of the suspending buffer medium, respectively; and $\zeta_{W}$ and $\zeta_{P}$ denote the zeta potentials of the channel wall and particle, respectively. The nonlinear EK phenomena considered here are dielectrophoresis (DEP) and nonlinear EP (EP_NL_). The expression of the DEP velocity of a spherical particle is as follows:

(3) $$v_{DEP}=\mu_{DEP}\nabla E_{rms}^{2}=\frac{r_{p}^{2}\varepsilon_{m}}{3\eta }Re\left[f_{CM}\right]\nabla E_{rms}^{2}$$

where $r_{p}$ is the particle radius; $Re\left[f_{CM}\right]$ is the real part of the Clausius–Mossotti factor, accounting for polarization effects; and $E_{rms}$ is the root mean square value of E. Regarding the velocity expressions for EP_NL_, the dimensionless applied field strength coefficient (β) and Peclet (*Pe*) and Dukhin (*Du*) numbers are required to identify the appropriate electric field dependence. There are only two limiting cases described using mathematical expressions for $v_{EP,NL}$, which are small *Pe* (*Pe* << 1) and high *Pe* (*Pe* >> 1). There are no well-established analytical expressions for the intermediate cases. The velocity expressions for these two limiting cases are given below [42,45,46]:

(4) $$v_{EP,NL}^{\left(3\right)}=\mu_{EP,NL}^{\left(3\right)}E^{3}{\hat{a}}_{E}\;\mathrm{for}\;\beta \sim 1,\;\mathrm{arbitrary}\;Du,\;\mathrm{and}\;Pe<<1\;(\mathrm{moderate field regime})$$

(5) $$v_{EP,NL}^{\left(3/2\right)}=\mu_{EP,NL}^{\left(3/2\right)}E^{3/2}{\hat{a}}_{E}\;\mathrm{for}\;\beta \;>\;1,\;Du<<1,\;\mathrm{and}\;Pe>>1\;(\mathrm{strong field regime})$$

where $\mu_{EP,NL}^{\left(n\right)}$ denotes the mobility of EP_NL_, which depends on E [41], and *n* denotes the dependence of $v_{EP,NL}$ on E as dictated by the operating conditions (see Table S1 in supplementary material). Considering these four distinct EK phenomena, the overall particle/cell velocity $(v_{P}$) in an iEK device as represented in Figure 1, is:

(6) $$v_{P}=v_{EO}+v_{EP,L}+{v_{DEP}+v}_{EP,NL}^{\left(n\right)}=\mu_{EO\;}E+\mu_{EP,L}\;E+\mu_{DEP}\nabla E^{2}+\mu_{EP,NL}^{\left(n\right)}E^{n}{\hat{a}}_{E}$$

The quality of each of the separations was quantified in terms of the separation resolution $\left(R_{s}\right)$, calculated as follows from the electropherograms:

(7) $$R_{s}=\frac{2(t_{R2,\;e}-t_{R1,e})}{W_{1}+W_{2}}$$

where W and $t_{R,e}$ denote the width of the peak at the base and the experimental retention time of each eluting species, respectively (Figure S1).

## 3. Materials and Methods

### 3.1. Fabrication of Microdevices

A cross-T iEK microchannel (Figure 1) was fabricated from polydimethylsiloxane (PDMS) using traditional soft lithography techniques [29]. After curing and gently detaching the PDMS casts of the microchannel, holes for inlet and outlet reservoirs were punched. The device fabrication process was completed by sealing the PDMS microchannel with a PDMS-coated glass wafer using corona treatment. The detailed dimensions of the microchannel, which was 30 µm deep, are provided in Figure 1.

### 3.2. Suspending Medium and Cell Samples

The suspending medium was a 0.2 mM solution of K_2_HPO_4_ with 0.05% (*v*/*v*) Tween-20. The pH and conductivity of the medium were adjusted to 7.1 ± 0.4 and 41.3 ± 5 µS/cm, respectively, by adding 0.1 N KOH solution. These conditions resulted in $\zeta_{W}$ and $\mu_{EO}$ of −60.1±3.7 mV and $4.7\pm 0.3\times 10^{-8}$ m^2^ V^−1^ s^−1^, respectively, which were measured experimentally using current monitoring experiments [47]. The two types of cells studied in this work were *E. coli* (ATCC 11775) and *S. cerevisiae* (ATCC 9763), whose properties are listed in Table 1. Standard procedures were used to culture and stain the cells using fluorescent SYTO dyes—Syto 11 (green) nucleic acid stain and Syto 85 (orange) nucleic acid stain (Thermo Fisher Scientific, Carlsbad, CA, USA) [7]. The values of $\zeta_{P}$, $\mu_{EP,L}$, and $\mu_{EP,NL}^{\left(3\right)}$ for both types of cells, possessing a negative surface charge, were independently measured using PTV experiments using a channel with a constant cross-section [48]. A binary mixture of these cells was injected into the iEK microchannel (Figure 1) using EK injection [49].

### 3.3. Equipment and Software

A high-voltage power supply (Model HVS6000D, LabSmith, Livermore, CA, USA) controlled using LabSmith Sequencer software version 1.167 was used to apply voltage to the microchannels through platinum wire electrodes (0.584 mm diameter and 1.5 cm length) labeled A–D (Figure 1). Experiments were recorded as videos using a Zeiss Axiovert 40 CFL (Carl Zeiss Microscopy, Thornwood, NY, USA) inverted microscope.

### 3.4. Experimental Procedure

Before experimentation, the microchannel was filled with the suspending medium to ensure stable EO flow. The cell mixture sample (~5 µL), comprising *E. coli* (5 ± 0.8 × 10^8^ cells/mL) and *S. cerevisiae* (1 ± 0.6 × 10^8^ cells/mL), was introduced into inlet reservoir A of the microchannel (Figure 1), after which the platinum electrodes were placed at each reservoir. A standard EK injection process [49], performed via sequential application of the voltages listed in Table 2, was used to inject the sample mixture to the post array region of the microchannel. The last step of the separation was determined by the elution of the cells from the channel. The fluorescence signal of each eluting cell species was captured at the end of the post array, as indicated in the interrogation window (Figure 1). Each of the two separations was repeated thrice to ensure reproducibility (Table S3).

### 3.5. Mathematical Modeling

Numerical models were built using COMSOL Multiphysics for predicting retention time ($t_{R,p}$), which was compared to the experimental retention time ($t_{R,\;e}$) of each cell type. The cell characteristics, assessed a priori [48], were utilized for predicting $t_{R,p}$ in the microchannel for each cell type with the appropriate stimulation voltage. Details on the mathematical model are included in the Supplementary Material (Figures S2–S5, Table S2).

## 4. Results and Discussion

### 4.1. Separation of Cells by Applying DC Signal

**Figure 2:**
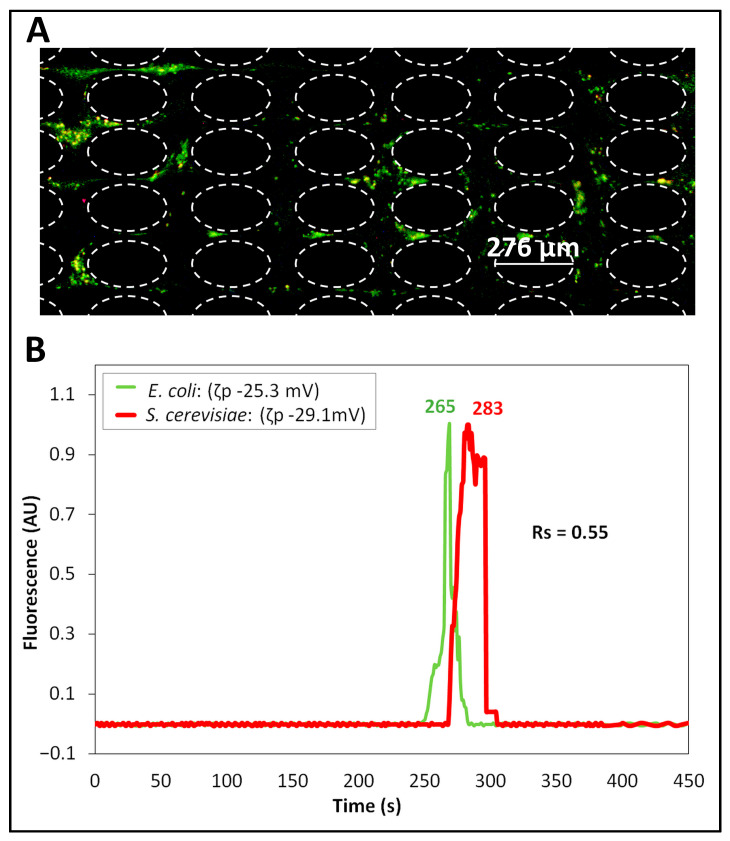
Separation (ID 1) of the cells performed by applying a DC voltage. (**A**) Image of the post array, where cell species are migrating while mixed with each other, i.e., the *E. coli* cells (labeled green) and the *S. cerevisiae* cells (labeled red) are interspersed. (**B**) Electropherogram of the separation built from the fluorescence signal of the cells recorded at the interrogation window. This separation was performed by applying a DC voltage with a 656 V magnitude, which was equivalent to the RMS value of the DC-biased AC voltage used for Separation ID 2.

To design the DC signal, the RMS value for the DC-biased AC voltage (used for Separation ID 2) was employed, as listed in Table 2. The experimental results from the separation of this binary cell mixture are shown in Figure 2. The cells appeared to be mixed with each other as they migrated across the post array (Figure 2A), indicating that no appreciable separation was taking place. This can be confirmed by the electropherogram in Figure 2B, featuring an overlap of the eluting peaks, with a poor separation resolution of $R_{s}$ = 0.55, indicating that the separation was incomplete ($R_{s}$ < 1.5). It can be noted from the electropherogram that although there was overlap in the elution of the cells, the *E. coli* cells reached the interrogation window slightly before the *S. cerevisiae* cells. This can be explained by the $\zeta_{P}$ and $\mu_{EP,L}$ values of the cells (Table 1). Good reproducibility, indicated by <10% deviation between repetitions, was obtained (Table S3). The deviation between model and experimental results, in terms of retention time, was below 19% (Table 3), illustrating that the model is a good tool for predicting the performance of this separation and can be used to design new separations. The COMSOL model was also used to study the effect of each individual EK phenomenon on the overall cell velocity across a cutline (Figure S3) between post constrictions, where the minimal effect of EP_NL_ on both cell species indicated that the separation was mainly in the linear regime (Figure S5A,B). In summary, the incomplete separation obtained by applying a DC voltage equivalent to the RMS value of the DC-biased AC voltage highlighted that for the cell mixture under consideration (Table 1), application of DC voltage alone was not sufficient to discriminate and separate the cells.

### 4.2. Separation of Cells by Applying DC-Biased Low-Frequency AC Signal

**Figure 3:**
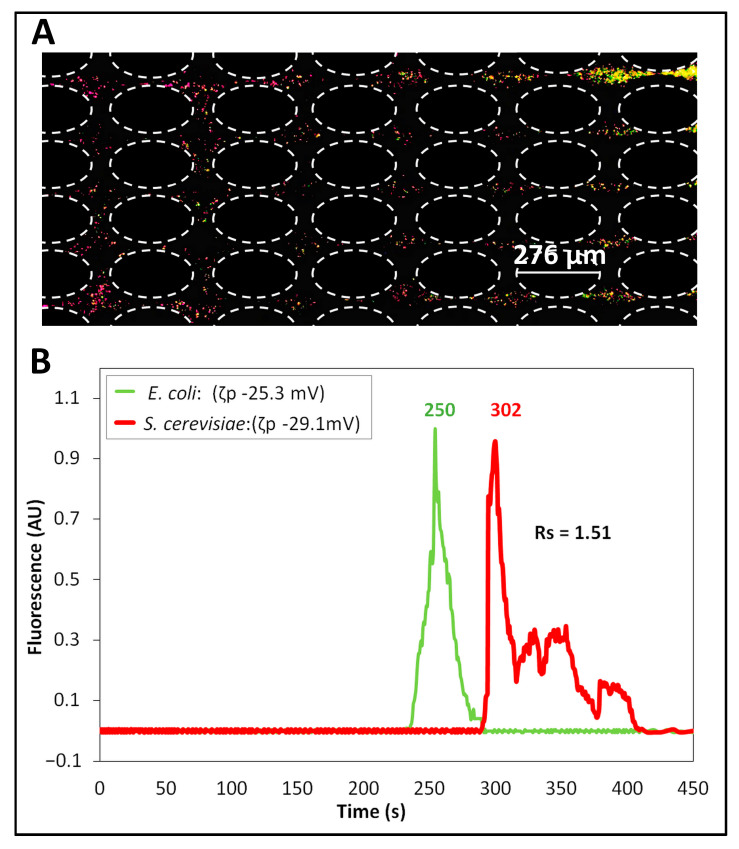
Separation (ID 2) of the cells performed by applying a DC-biased AC voltage. (**A**) Image of the post array, where the cells begin to form “zones” of cells, illustrating that the *E. coli* cells (labeled green) are moving ahead of the *S. cerevisiae* cells (labeled red). (**B**) Electropherogram of the separation obtained by analyzing the fluorescence signal of the cells, recorded at the interrogation window. This separation was performed by applying a DC-biased AC voltage having 500 V DC bias and 600 V peak amplitude at 0.4 Hz.

The second separation was carried out by applying a DC-biased low-frequency AC electric voltage, which was carefully selected from COMSOL simulations to have a difference between the predicted retention times of the cells of at least 30 s. Our previous work established that a difference of at least 30 s (Δ$t_{R,p}$ > 30 s) is required for a separation to be successful [43]. The identified voltage was a 500 V DC-biased 600 V peak amplitude at 0.4 Hz frequency. This AC voltage was the one “mimicked” by the DC voltage used in Separation ID 1. Since successful separation of microparticles and cells has been previously reported at 0.4 Hz [29,43], this was chosen as the frequency for these separation experiments. The experimental results of this separation are shown in Figure 3. The image in Figure 3A shows the formation of “zones” of *E. coli* and *S. cerevisiae* cells as they migrated across the insulating post array. The green-labeled *E. coli* cells were migrating ahead of the red-labeled *S. cerevisiae* cells. This result was as expected from the $\zeta_{P}$ and $\mu_{EP,L}$ values of the cells (Table 1), since *E. coli* cells have lower magnitudes of $\zeta_{P}$ and $\mu_{EP,L}$, allowing these cells to migrate faster towards the outlet and elute first. The electropherogram of this separation is shown in Figure 3B with a separation resolution of $R_{s}$ = 1.51, indicating a complete separation with well-resolved peaks. It is important to acknowledge the non-Gaussian shape of the peaks, which is the result of the back-and-forth movement of the cells due to the AC signal application. Good reproducibility of <10% was obtained between experimental repetitions for each cell species (Table S3). As conducted with Separation ID 1, the COMSOL model was used to study the effects of the four distinct EK phenomena on the overall cell velocity (Figure S5C,D). For *S. cerevisiae* cells, there was a moderate effect of EP_NL_ on the overall cell velocity, which was beneficial for the separation. The deviation between model and experimental results, in terms of retention time, was below 12% (Table 3), indicating good agreement and reiterating the applicability of the model for designing new separation processes. Potential causes of the deviations between modeling and experimental results are EK injection bias during the sample injection process, local electric field distortions caused by the particles themselves, and particle–particle interactions, since none of those are accounted for in the model [49].

### 4.3. Comparison of the Separation Effectiveness Obtained with the DC Voltages and with the DC-Biased Low-Frequency AC Voltages

**Figure 4:**
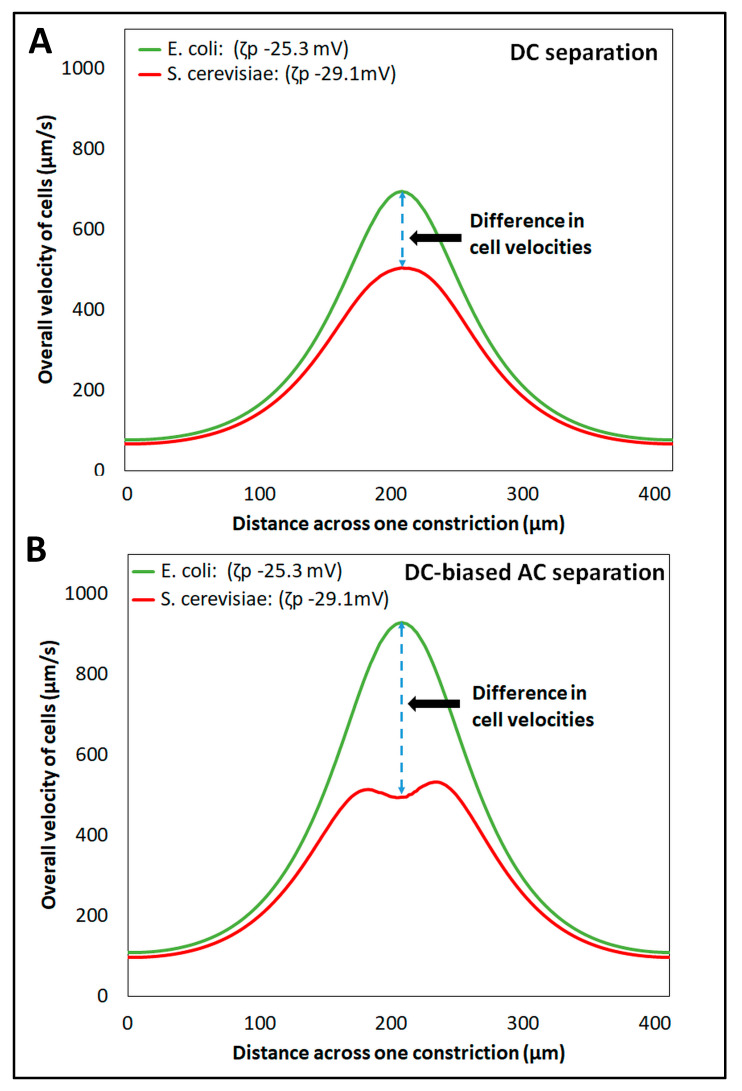
Prediction of the overall cell velocities across a cutline (shown in Figure S3) between two posts’ constrictions, with the two types of separation—(**A**) Separation ID 1, employing a DC potential at 656 V, and (**B**) Separation ID 2, employing a DC-biased AC voltage having 500 V DC bias and 600 V peak amplitude at 0.4 Hz, respectively.

The separation of cells is mainly governed by the differences in the overall cell velocity, as expressed in Equation (6), which depends on the individual velocity components of the four EK phenomena, as shown in Equations (1)–(5). The magnitudes of all EK phenomena depend on the electric field and the position of the cells in the iEK channel. For both the separations (Separation IDs 1–2), the nonuniform electric field across the iEK channel, caused by the presence of insulating posts, exerted a combination of linear and nonlinear EK effects on the cells, which affected their overall velocities (Figure S5). For Separation ID 1, employing a DC potential, whose only relevant characteristic was its magnitude, the electric field distribution did not vary with time; therefore, its maximum magnitude was not time-dependent either. In contrast, for Separation ID 2, employing a DC-biased AC potential, which had a temporal component, the electric field distribution was time-dependent, and the maximum electric field magnitude was reached at the time of peak amplitude application. The prediction of overall cell velocity for both *E. coli* and *S. cerevisiae* cells across a cutline between two posts’ constrictions (cutline shown in Figure S3), at the maximum magnitude of the electric field, for both separations (Separation IDs 1–2) is shown in Figure 4. The difference between the two distinct cell velocities for Separation ID 1 (Figure 4A), employing a DC voltage, was much smaller than that obtained with Separation ID 2 (Figure 4B), employing a DC-biased AC potential. This larger difference between the two distinct overall cell velocities obtained with the DC-biased AC potential at the maximum electric field magnitude, at the time of peak amplitude application, increased the discrimination capability between the two cell species. Therefore, the application of the DC-biased AC potential resulted in a complete separation ($R_{s}$ = 1.51), compared to the incomplete separation ($R_{s}$ = 0.55) obtained by applying the DC potential.

## 5. Conclusions

Presented here is the separation of a binary mixture of cells (*E. coli* and *S. cerevisiae*) in an iEK microchannel employing two distinct electric stimulations: a DC voltage and a low-frequency DC-biased AC voltage. The magnitude of the DC voltage was designed to be equivalent in magnitude to the RMS of the DC-biased AC voltage. Mathematical modeling with COMSOL was used to guide experimentation (selection of appropriate electric voltages to apply) and to gain understanding of the effect of the four distinct different EK phenomena influencing cell migration behavior. The iEK separation employing the DC voltage had poor performance, with a separation resolution of $R_{s}$ = 0.55, while the separation with the DC-biased AC voltage resulted in a complete separation, with $R_{s}$ = 1.51. The two distinct separations had good experimental reproducibility, with deviations below 10% between experimental repetitions. Good agreement was also obtained between modeling and experimental results, with deviations below 19% for all cases. This is the first study to compare the separation performance of a binary mixture of cells in an iEK device by applying two types of potential: a DC potential and a DC-biased AC potential. The results from this work highlight the importance of the type of electric stimulation being used for the same separation in the same iEK device geometry. The main finding from this study is that the cell mixture under consideration remains mixed and does not separate when using a DC voltage but it is effectively separated using a DC-biased AC voltage.

This follow-up study to our previous study on fine-tuning low-frequency AC voltages to improve the separation resolution of mixtures of microparticles demonstrates the discriminatory capability of AC-iEK systems to enable cell separations, which cannot be achieved with their DC-iEK counterpart. The ability of AC voltages to effectively discriminate cells opens up the potential for future research to separate microscopic entities of interest using an iEK system stimulated with AC potentials at different low frequencies and investigate the effect of frequency on cell separations. Future contributions to this field will assess the application of AC-iEK systems to separate cells or particles with more similar characteristics, evaluate the influence of the microchannel wall on the experimental results of separation, and extend the applications of these systems to complex mixtures containing three or more distinct target analytes.

## Figures and Tables

**Table 1:** Characteristics of the cells used in this study.

Cell ID	Size (µm)	$\zeta_{P}$ (mV)	$\mu_{EP,L}$ × 10^−8^ (m^2^V^−1^s^−1^)	$\mu_{EP,NL}^{\left(3\right)}$ × 10^−18^ (m^4^V^−3^s^−1^)
*E. coli* (ATCC 11775)	3.2 ± 0.3 long 1.1 ± 0.2 wide	−25.3 ± 2.1 ^2^	−1.97 ± 0.1 ^2^	−2.1 ± 0.1 ^1,2^
*S. cerevisiae* (ATCC 9763)	7.0 ± 0.7 diameter	−29.1 ± 3.7 ^2^	−2.26 ± 0.3 ^2^	−7.6 ± 1.5 ^1,2^

^1^ Average approximation obtained through analytical curve fitting of cubic dependence of EP_NL_ velocity on E. ^2^ Characteristics such as $\zeta_{P}$, $\mu_{EP,L}$, and $\mu_{EP,NL}^{\left(3\right)}$ mobilities depend on the suspending medium and are specific to these cell–fluid systems.

**Table 2:** Voltage conditions used for EK injection and separation of the cells.

Separation ID	Description	Step	Run Time (s)	Applied Voltage (V)			
				A	B	C	D
1	Separation using DC potential	Loading (DC)	10	500	300	0	500
		Gating (DC)	5	1000	1000	1000	0
		Injection (DC)	5	0	1000	0	0
		Separation (DC)	450	200	656	200	0
2	Separation using DC-biased AC potential	Loading (DC)	10	500	300	0	500
		Gating (DC)	5	1000	1000	1000	0
		Injection (DC)	5	0	1000	0	0
		Separation (DC bias + AC)	450	200	$500\;(\mathrm{DC})+600\;(V_{p})$ @ 0.4 Hz	200	0

**Table 3:** Assessment of the separations in terms of $R_{s}$, comparison of $t_{R,p}$ and $t_{R,e}$, and reproducibility between separation experiments.

Separation ID	Cell ID	$R_{s}$	$\mathrm{COMSOL}\;\mathrm{Predicted}\;t_{R,p}$ (s)	$\mathrm{Average}\;\mathrm{of}\;\mathrm{Experimental}\;t_{R,e}$(s)	$\mathrm{Deviation}\;\mathrm{between}\;t_{R,p}$$\;\mathrm{and}\;t_{R,e}$(%)	Experimental Deviation between Repetitions (%)
1	*E. coli*	0.55	197.0	239.7	17.8	7.8
	*S. cerevisiae*		224.2	274.3	18.3	6.6
2	*E. coli*	1.51	228.6	241.7	5.4	2.5
	*S. cerevisiae*		261.3	295.7	11.6	2.6

## Data Availability

Data are contained within the article and Supplementary Materials.

## Supplementary Materials

The following supporting information can be downloaded at https://www.mdpi.com/article/10.3390/mi14122239/s1, Table S1: Values of the parameters used to analyze the moderate field regime, cubic dependence (E^3^); Table S2: COMSOL model information; Table S3: Experimental repetitions of separations; Figure S1: Illustration of peaks in an electropherogram; Figure S2: Depiction of domains and boundaries used in computational model; Figure S3: Representation of the cutline constructed in COMSOL Multiphysics to determine predicted retention time for the cells to migrate across the insulating post array; Figure S4: Curve-fitting results using a Fourier series expansion of the E-field profile; Figure S5: Prediction of the overall and individual cell velocities of the four EK phenomena across the cutline, with the two types of separation (Separation IDs 1–2); Video S1: Separation by applying DC potential; Video S2: Separation achieved by applying DC-biased AC potential.
