# Supplementary material for: Assessing the Discriminatory Capabilities of iEK Devices under DC and DC-Biased AC Stimulation Potentials
Source: Micromachines (Basel). 2023 Dec 14;14(12):2239. doi: 10.3390/mi14122239 (PMC10745336; doi:10.3390/mi14122239)
Supplement: Supplementary file 1 [file micromachines-14-02239-s001.zip › Supplementary material.pdf]

Supplementary Material

Assessing the discriminatory capabilities of iEK devices under DC and DC-biased AC stimulation potentials

Nuzhet Nihaar Nasir Ahamed[a], Carlos A. Mendiola-Escobedo[b], Victor H. Perez-Gonzalez[b,*] and Blanca H. Lapizco-Encinas[a,*]

[a] Microscale Bioseparations Laboratory and Biomedical Engineering Department, Rochester Institute of Technology, 160 Lomb Memorial Drive, Rochester, New York, 14623, United States.

[b] School of Engineering and Sciences, Tecnologico de Monterrey, Monterrey, Nuevo Leon 64849, Mexico.

Correspondence should be addressed to the following authors:
Victor H. Perez-Gonzalez (PhD)
Email: vhpg@tec.mx

Blanca H. Lapizco-Encinas (PhD)
Email: bhlbme@rit.edu

Contents

ASSESSMENT OF THE NONLINEAR ELECTROPHORETIC VELOCITY UNDER THE MODERATE ELECTRIC FIELD REGIME: INCLUDES TABLE S1 2

ILLUSTRATION OF PEAKS IN AN ELECTROPHEROGRAM: INCLUDES FIGURE S1 2

COMSOL MODEL INFORMATION: INCLUDES FIGURE S2, AND TABLE S2 3

ESTIMATION OF THE PREDICTED RETENTION TIME USING COMSOL MODEL DATA: INCLUDES FIGURE S3 AND FIGURE S4. 4

Construction of horizontal cutline across the insulating post array constrictions: 4

Details on algorithm used for predicting $t_{R,p}$: 4

COMPARISON OF THE VELOCITY MAGNITUDES OF ALL FOUR EK PHENOMENA: INCLUDES FIGURE S5. 7

REPRODUCIBILITY BETWEEN REPETITIONS: INCLUDES TABLE S3 8

REFERENCES 8

Assessment of the nonlinear electrophoretic velocity under the moderate electric field regime: includes Table S1

The expression of the three dimensionless parameters (β, Du and Pe) utilized to identify the appropriate field regime of the nonlinear electrophoretic velocity are [1–3]:

$$\beta = \frac{Ea}{\varphi} \quad \text{(S1)}$$

$$Du = \frac{K^{\sigma}}{K_m a} \quad \text{(S2)}$$

$$Pe = \frac{a|(\boldsymbol{v}_{EP,L} + \boldsymbol{v}_{EP,NL})|}{D} \quad \text{(S3)}$$

where E is the applied electric field magnitude, a is the particle radius, φ is the thermal voltage, $K^{\sigma}$is surface conductivity, K_m is bulk conductivity, $\boldsymbol{v}_{EP,L}$ and $\boldsymbol{v}_{EP,NL}$ are the linear and nonlinear electrophoretic particle velocity, respectively, and D is the diffusion coefficient.

Table S1. Values of the parameters used to analyze the moderate field regime, cubic dependence ($\mathbf{E^3}$).

Separation ID	Cell ID – Label color	β	Du	Pe	E used for parameters estimation (V/cm)	Dependence of nonlinear EP with E
1	*E. coli* - Green	0.3	0.09	0.1	100	Moderate ($\mathbf{E}^3$)
	S. cerevisiae - Red	0.8	0.02	0.3	50	Moderate ($\mathbf{E}^3$)

Illustration of peaks in an electropherogram: includes Figure S1

The illustration of an electropherogram showing width of the peak at the base (W) and the experimental retention time of each eluting species ($t_{R,e}$) is shown in Figure S1.

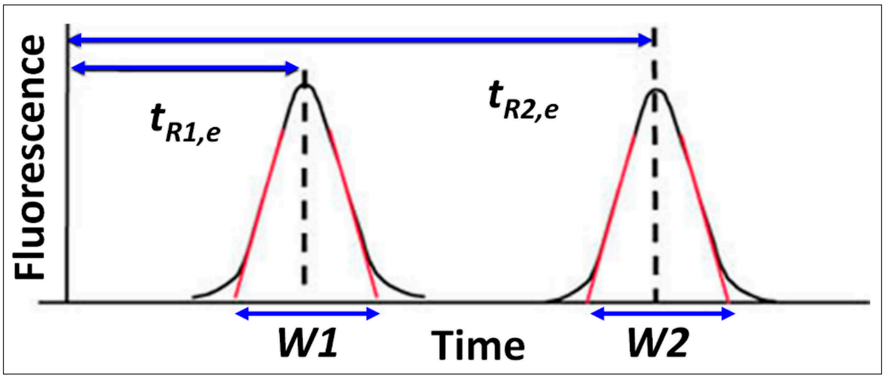

Figure S1. Illustration of peaks in an electropherogram constructed by plotting fluorescence of eluting species with respect to time, $W1$ and $W2$ indicate the width of the peaks at the peak base, and $t_{R1,e}$ and $t_{R2,e}$ denote the retention time of eluting species 1 and 2, respectively.

COMSOL model information: includes Figure S2, and Table S2

COMSOL *Multiphysics* was used to build a 2D computational model for estimating the electric field distribution across the iEK device. As channel depth changes can be ignored, this 2D model is an appropriate choice for the modeling [4]. The Electric Currents module within COMSOL *Multiphysics* was utilized for solving the Laplace equation. All material properties required for the model were acquired from the COMSOL Material Library, and the relative permittivity and conductivity of the fluid suspending medium (ε_r) were set to 78.4 and 4.07 x 10^{-3} S/m (40.7 µS/cm), respectively. **Figure S2** depicts the different domains and boundary conditions used, and **Table S2** lists these conditions as equations. Maximum and minimum element sizes for free triangular meshes were 130 µm and 0.261 µm, respectively.

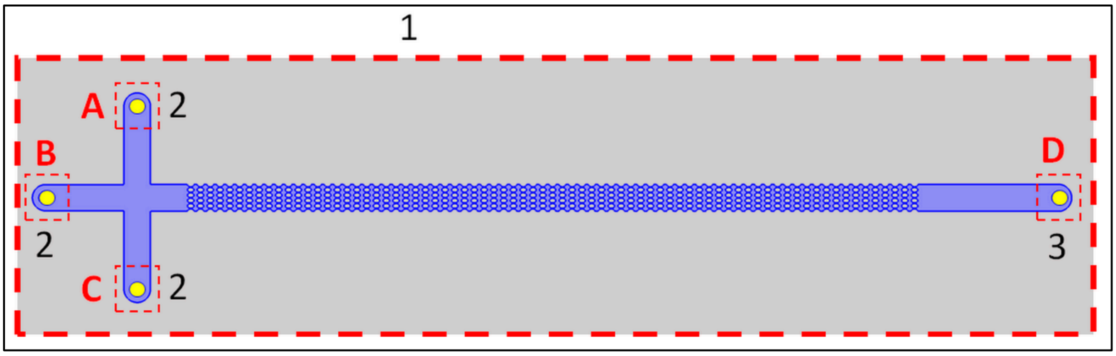

Figure S2. Depiction of the domains and boundaries used in the computational model. The red dotted boxes represent the boundaries used in the computational model. The labels A, B, C, and D indicate electrodes used in the EK injection and separation process. Each colored area shows each of the different domains employed: the gray color shows the PDMS domain, the blue color illustrates the fluid suspending medium field, and the yellow color indicates the platinum electrodes. Device dimensions are shown in **Figure 1** in the manuscript. The numbers 1, 2, and 3 are used to exhibit the boundaries listed in **Table S2**. Boundary 1 represents the outer PDMS surface; boundary 2 describes the three reservoirs where electric potentials are applied: A, B, and C. Boundary 3 is the ground electrode D.

Table S2. COMSOL model information: Domain and Boundary conditions defined in the model. Domains are displayed in **Fig. S2**. The labels A, B, C, and D, are the labels used to indicate the electrodes for the EK injection and separation process, as reported in **Table 2** of the manuscript.

**Domain conditions**		
**Domain type**	**Element region & color**	**Definition***
Current conservation	PDMS domain (channel walls and insulating posts), gray color	$\nabla \cdot \mathbf{J} = -\nabla \cdot \left(\left(\sigma + \varepsilon_0 \varepsilon_r \frac{\partial}{\partial t}\right) \mathbf{E}\right)$; where $\mathbf{E} = -\nabla V$
	Fluid suspending medium domain, blue color	
	Platinum electrodes domain, yellow color	
Initial values	PDMS domain (channel walls and insulating posts), gray color	$V_0 = 0$
	Fluid suspending medium domain, blue color	
	Platinum electrodes domain, yellow color	
**Boundary conditions**		
**Boundary condition type**	**Element number**	**Definition**
Electric insulation	1	$\mathbf{n} \cdot \mathbf{J} = 0$
Electric potential	2	$V_A = V_{applied,A}$; $V_B = V_{applied,B}$; for Separation ID 1; $V_B(t) = V_{DC} + V_p \sin(\omega t)$; for Separation ID 2;

$V_C = V_{applied,C}$;
$V_D = V_{applied,D}$;

*In this table, V represents the electric potential, for Separation ID 1, employing DC potential featuring only magnitude, is independent of time (t), for Separation ID 2, featuring a temporal component, V_p is the peak amplitude of the applied electric potential in DC-biased (V_{DC}) AC signal, **J** is the electric current density and $\omega = 2\pi f$, with f being the AC frequency. The variables ε_0 and ε_r represent the permittivity of the vacuum and the relative permittivity of each given domain, respectively. The permittivity of a given domain is $\varepsilon = \varepsilon_0\varepsilon_r$.

Estimation of the predicted retention time using COMSOL model data: includes Figure S3 and Figure S4.

Construction of horizontal cutline across the insulating post array constrictions:

A horizontal cutline, 411 µm long, was constructed across two post constrictions, and is set in the middle of the post array, as shown in **Figure S3**. The total retention time ($t_{R,p}$) for each particle to migrate across the cutline was predicted for each particle using the electric field data measured over the cutline. In this way, using **Eq. (6)** from the main article, overall cell velocity can be predicted directly from the electric field generated within the device. As this data is provided as a set of points given by the selected mesh in COMSOL, it should be appropriate to fit an analytical curve to consider the information of the regions between points and attain a better prediction. After determining this information for one constriction, the same profile is considered for the next constrictions until the particle position is greater than or equal to the overall length of the array. This lays the foundation for the construction of an algorithm to predict $t_{R,p}$.

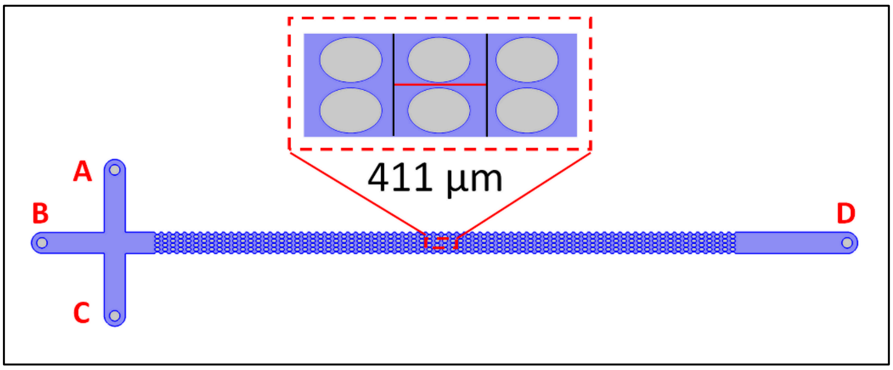

Figure S3. Representation of the cutlines constructed in COMSOL *Multiphysics* to determine predicted retention time for the cells to migrate across the insulating post array. The length of the cutline considered is 411 µm, as it begins and terminates across the center of two adjacent lateral openings between the rows of insulating posts. Since posts are 276 µm wide, and each lateral constriction is 135 µm, the total cutline length is 67.5 µm + 276 µm + 67.5 µm = 411 µm.

Details on algorithm used for predicting $t_{R,p}$:

The objective of this section is to develop an algorithm to predict particle position along the microchannel illustrated in **Figure S3**. This estimation will be referred as $\hat{r}_p$. Particle speed has two types of dependencies with the electric field (E): one linked to its magnitude, related to electroosmotic (EO) flow, linear and nonlinear electrophoresis (EP); and one linked to $\nabla|E^2|$, related to dielectrophoresis (DEP). Each response can be associated to a mobility (μ), such that:

$$v_p = \left(\mu_{EO} + \mu_{EP}^{(1)}\right) E + \mu_{EP}^{(n)} E^n + \mu_{DEP}\left|\nabla|E^2|\right|. \quad \text{(S4)}$$

In this way, the use of an analytic curve is crucial to obtain the best approximation possible to the E or $\nabla|E^2|$ profile, dependent on both position and time. Such distributions have two properties that must be satisfied in the fitted curve: (a) at least within the post section of the channel, it is periodic and (b) the derivative with respect to x is continuous in the boundaries between constrictions. Properties that are naturally satisfied by their Fourier series expansion, with the period being defined as the length of one constriction (L). In this way, the predicted E-field ($\hat{E}$) can be defined as:

$$\hat{E}(x) = a_0 + \sum_{n=1}^{N} \left[a_n \sin\left(\frac{2n\pi}{L}x\right) + b_n \cos\left(\frac{2n\pi}{L}x\right)\right], \tag{S5}$$

in which

$$a_0 = \frac{1}{L}\int_0^L E(x)\, dx, \tag{S6}$$

$$a_n = \frac{2}{L}\int_0^L E(x) \sin\left(\frac{2n\pi}{L}x\right)\, dx, \tag{S7}$$

$$b_n = \frac{2}{L}\int_0^L E(x) \cos\left(\frac{2n\pi}{L}x\right)\, dx, \tag{S8}$$

On the other hand, the predicted $\left|\nabla|E^2|\right|$ profile ($\hat{D}$) will be obtained by directly differentiating the Fourier series expansion of $E^2(x)$, to use a single set of points and not loose any information by performing a numerical derivative. Since the problem is reduced to the x-axis, the gradient becomes one dimensional. In this way:

$$\hat{D}^{(2)}(x) = \left|\widehat{\nabla|E^2|}\right| = \frac{d}{dx}\left\{A_0 + \sum_{n=1}^{N}\left[A_n \sin\left(\frac{2n\pi}{L}x\right) + B_n \cos\left(\frac{2n\pi}{L}x\right)\right]\right\}, \tag{S9}$$

in which the superscript (2) references that this quantity is related to the gradient of the E-field squared and:

$$A_0 = \frac{1}{L}\int_0^L E^2(x)\, dx, \tag{S10}$$

$$A_n = \frac{2}{L}\int_0^L E^2(x) \sin\left(\frac{2n\pi}{L}x\right)\, dx, \tag{S11}$$

$$B_n = \frac{2}{L}\int_0^L E^2(x) \cos\left(\frac{2n\pi}{L}x\right)\, dx. \tag{S12}$$

After carrying out the derivative in eqn. (S9), $\hat{D}^{(2)}(x)$ becomes:

$$\hat{D}^{(2)}(x) = \frac{2n\pi}{L}\sum_{n=1}^{N}\left[A_n \cos\left(\frac{2n\pi}{L}x\right) - B_n \sin\left(\frac{2n\pi}{L}x\right)\right], \tag{S13}$$

which satisfies the use of the same coefficients of the Fourier expansion of $E^2(x)$ and the use of a single set of points to carry out both curve fittings. Also, given its definition, the term A_0 is of no use for this expansion.

Using this logic, we will define a $\hat{D}^{(1)}$ profile, related to the gradient of the E-field. Referencing eqn. (S13) one can deduct that such field will be:

$$\hat{D}^{(1)}(x) = \frac{2n\pi}{L}\sum_{n=1}^{N}\left[a_n \cos\left(\frac{2n\pi}{L}x\right) - b_n \sin\left(\frac{2n\pi}{L}x\right)\right]. \tag{S14}$$

Due to the discrete nature of the extracted E-field from COMSOL, the integrals illustrated in eqns. (S5)-(S7) and (S11)-(S12) must be carried out numerically. For this, the *scipy* library was used in a Python code to apply the appropriate Simpson rule to the E-field data. This same code calculated the Fourier coefficients of the expansion and stored them to be used later in the analytic prediction $\hat{E}(x)$. It should be noted that, in eqns. (S5) and (S13), the Fourier series is truncated on the N-th term, due to the E-profile being a discrete set of points. As frequency increases, the number of points to get a well-defined sinusoidal function increases as well. As it is hard to define a truncation term given the number of points of the raw data, an algorithm was implemented in the mentioned code to expand the distribution until the mean squared error is no longer minimized.

Because of the direct proportionality relation between the E-field and the applied voltage in the channel (V), it is asserted that $\frac{E_1}{V_1} = \frac{E_2}{V_2}$. With this, the curve fitting of the E-field can be performed for only one voltage input and then rescale it as necessary. Due to the properties of the analyzed channels, this process was performed for terminals A and C together and terminal B individually as terminals A and C provide an extra DC-bias signal and terminal B provides either a DC or AC+DC signal. For this study, a voltage $V_0 = 100\,V$ was used for terminal B and the distribution generated by terminals A and C was directly analyzed. Results of the performed curve fittings for both E and $\left|\nabla|E^2|\right|$ are illustrated in **Figure S4**.

For this study, it was shown with the quantities illustrated in **Table S1** that the nonlinear EP order was moderately cubic. With this, and the analytic curve fitting of the E-field data, the predicted particle speed is defined as:

$$\hat{v}_p(t,x) = \left(\mu_{EO} + \mu_{EP}^{(1)}\right)\left[\hat{E}_{A,C}(x) + V_B(t)\hat{E}_B(x)\right] + \mu_{EP}^{(3)}\left[\hat{E}_{A,C}(x) + V_B(t)\hat{E}_B(x)\right]^3 + \mu_{DEP}\hat{H}(x,t)\,, \tag{S14}$$

with

$$\hat{H}(x,t) = \hat{D}_{A,C}^{(2)}(x) + V_B^2(t)\hat{D}_B^{(2)}(x) + 2V_B(t)\left[\hat{E}_{A,C}(x)\hat{D}_B^{(1)}(x) + \hat{E}_B(x)\hat{D}_{A,C}^{(1)}(x)\right], \tag{S15}$$

which is obtained when $\left|\nabla\left|\left[\hat{E}_{A,C}(x) + V_B(t)\hat{E}_B(x)\right]^2\right|\right|$ is expanded. Additionally:

$$V_B(t) = \frac{1}{V_0}\left[V_{DC} + V_p\sin(\omega t)\right]. \tag{S16}$$

Finally, an estimation of retention time can be obtained by solving the differential equation:

$$\frac{d}{dt}\hat{r}_p(t) = \hat{v}_p(t,x), \tag{S17}$$

which can be carried out with the use of the 4th order Runge-Kutta method implemented in the previously mentioned code. In this way, $t_{R,p}$ is extracted when the condition $\hat{r}\left(t_{R,p}\right) \geq L$ is met. The temporal step used to solve eqn. (S17) is the one from the function generator used in experiments, which is of 0.06 s.

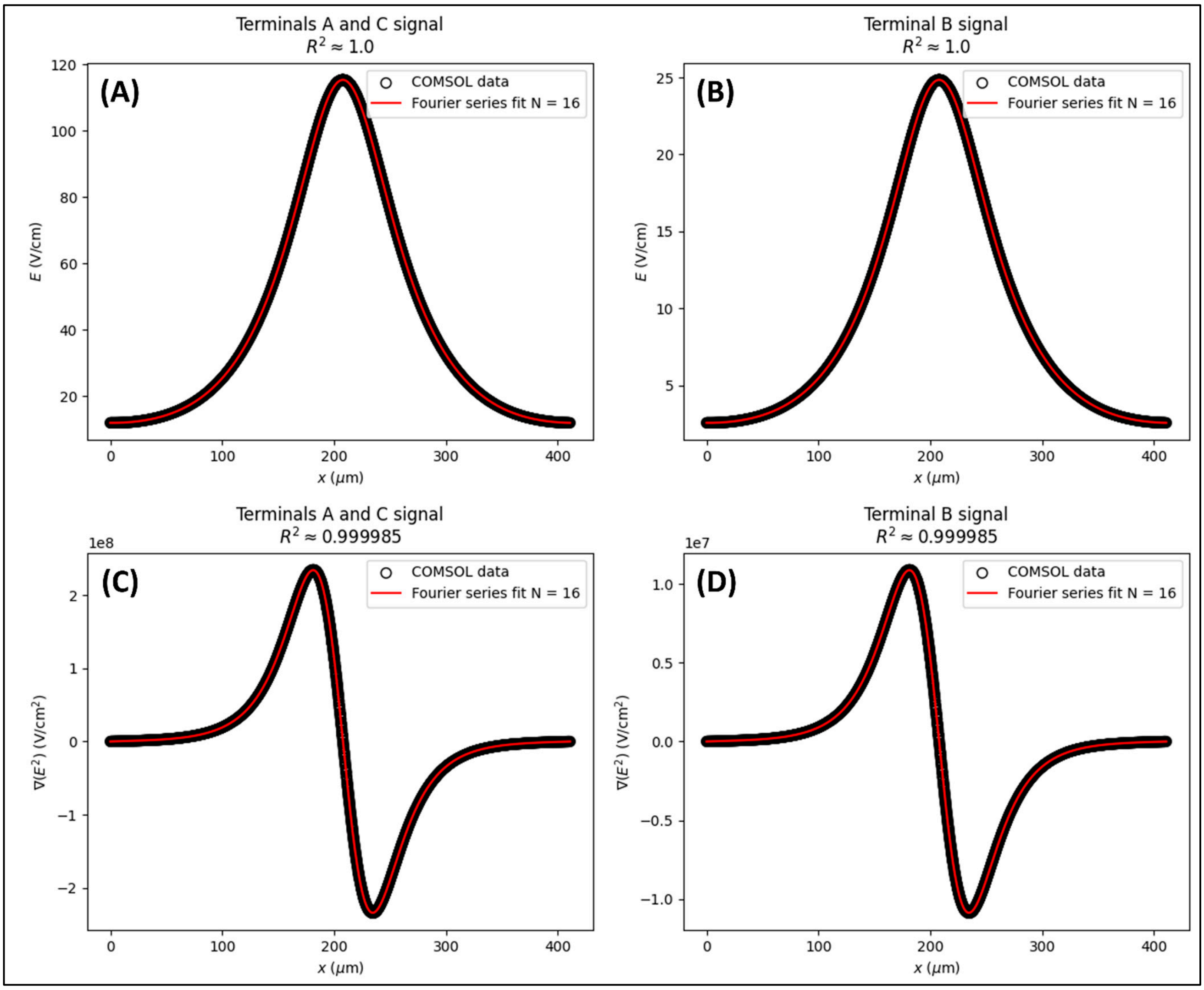

Figure S4. Curve fitting results using a Fourier series expansion of the E-field profile. In scattered marking the E-field provided by COMSOL is plotted and the red line contains the curve fitting attained with the Fourier series expansion. For both data sets, the truncation element was found to be 16. The benchmark selected to define a good fit was the determination coefficient R^2 rounded to six decimals. **(A, C)** Contains the extracted data of the E-field and $|\nabla|E^2||$ with their respective Fourier series expansions for terminals A and C, **(B, D)** contains the extracted data of the E-field and $|\nabla|E^2||$ with their respective Fourier series expansions for terminal B.

Comparison of the velocity magnitudes of all four EK phenomena: includes Figure S5.

Depiction of particle velocities over the cutline is shown in **Figure S5**, where the effect that different types of electric stimulation potentials (DC and DC-biased AC) used for separation have on particle velocities is investigated. Based on the prediction of individual particle velocities resulting from each one of the four electrokinetic phenomena, it can be observed that both separations (Separation IDs 1-2) are mainly in the linear EK regime, as linear EO and linear EP are the main EK phenomena contributing to the overall particle velocities. It is

also important to note that at the voltages selected (**Table 2** in the manuscript), the magnitude of the nonlinear EK effects of DEP and $EP_{NL}^{(3)}$ was kept low/moderate, thus, enabling the separation to take place by mainly exploiting linear EP effects.

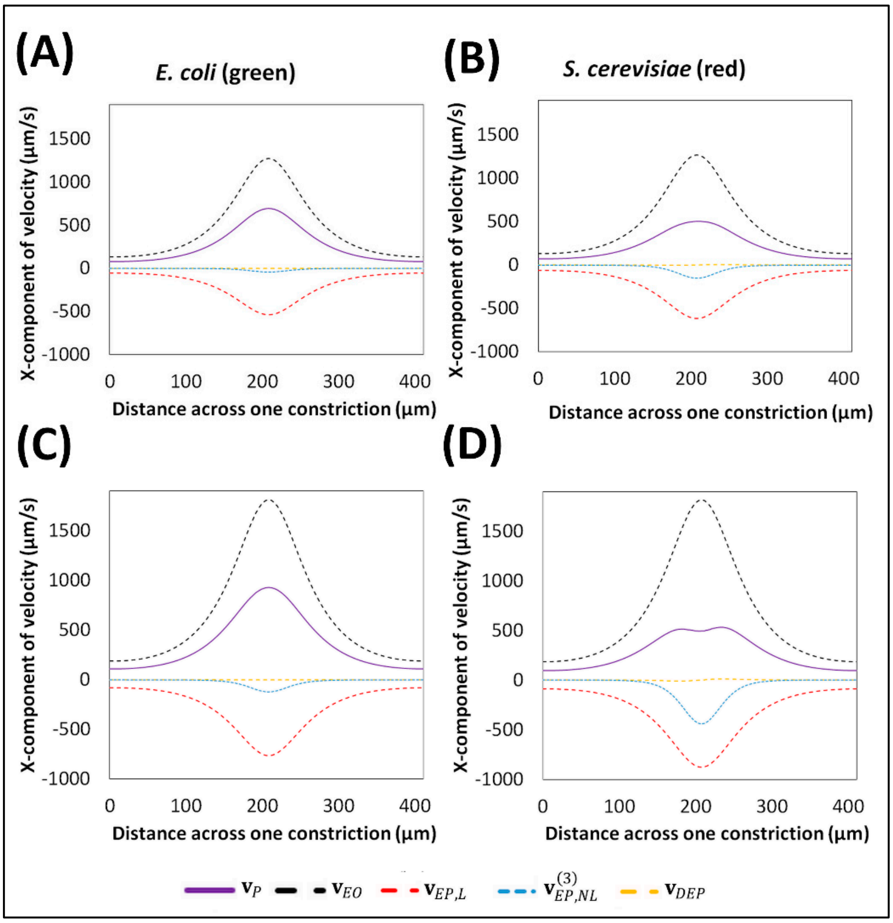

Figure S5. Prediction of the overall and individual cell velocities exerted by the four EK phenomena across the cutline, with the two types of separation (Separation IDs 1-2). Individual cell velocities exerted by four EK phenomena across a constriction, along the cutline in **Figure S3**, for *E. coli* (green) and *S. cerevisiae* (red) cells, in: (A,B) DC separation employed at 656 V and (C, D) DC-biased AC separation employed at 500 V DC bias, 600 V peak amplitude at 0.4 Hz, respectively.

Reproducibility between repetitions: includes Table S3

Table S3. Values of retention times for three distinct experimental repetitions of each separation. Separation ID 1 was performed by applying a DC signal and Separation ID 2 was performed with a DC-biased low-frequency AC signal.

Separation ID	Cell ID – label color	$t_{R,e}$ Repetition 1 (s)	$t_{R,e}$ Repetition 2 (s)	$t_{R,e}$ Repetition 3 (s)	Average (s)	Range of stdev (%)
1	*E. coli* - Green	265	220	234	240	8

	S. cerevisiae - Red	283	249	291	274	7
2	*E. coli* - Green	250	236	239	242	3
	S. cerevisiae - Red	302	300	285	296	3

References

1. Schnitzer, O.; Zeyde, R.; Yavneh, I.; Yariv, E. Weakly Nonlinear Electrophoresis of a Highly Charged Colloidal Particle. *Phys. Fluids* **2013**, *25*, 052004, doi:10.1063/1.4804672.
2. Schnitzer, O.; Yariv, E. Nonlinear Electrophoresis at Arbitrary Field Strengths: Small-Dukhin-Number Analysis. *Phys. Fluids* **2014**, *26*, 122002, doi:10.1063/1.4902331.
3. Cobos, R.; Khair, A.S. Nonlinear Electrophoretic Velocity of a Spherical Colloidal Particle. *J. Fluid Mech.* **2023**, *968*, A14, doi:10.1017/jfm.2023.537.
4. Gallo-Villanueva, R.C.; Perez-Gonzalez, V.H.; Cardenas-Benitez, B.; Jind, B.; Martinez-Chapa, S.O.; Lapizco-Encinas, B.H. Joule Heating Effects in Optimized Insulator-Based Dielectrophoretic Devices: An Interplay between Post Geometry and Temperature Rise. *Electrophoresis* **2019**, *40*, doi:10.1002/elps.201800490.
